# Application of SPECT/CT muscle imaging in determining the involved muscles in cervical dystonia

**DOI:** 10.1007/s12149-026-02178-0

**Published:** 2026-03-11

**Authors:** Ting-Ting Shen, Quan-Peng Wang, Qi-Lin Zhang, Qian-Chang Wu, Jun Wang, Jing Liu, Bao-Ming Mi, Wei-Feng Luo

**Affiliations:** 1 Department of Neurology, The Second Affiliated Hospital of Jiaxing University, Jiaxing, 314000 Zhejiang China; 2 Department of Nuclear Medicine, Affiliated Hospital of Jiangnan University, Wuxi, 214000 Jiangsu China; 3 Department of Neurology and Suzhou Clinical Research Center of Neurological Disease, The Second Affiliated Hospital of Soochow University, Suzhou, 215004 Jiangsu China; 4 Department of Nuclear Medicine, The Second Affiliated Hospital of Soochow University, Suzhou, 215004 Jiangsu China; 5 Jiangsu Key Laboratory of Neuropsychiatric, Diseases of Soochow University, Suzhou, 215004 Jiangsu China

**Keywords:** Cervical dystonia, SPECT/CT, SUVmax, Botulinum toxin A

## Abstract

**Purpose:**

This study examines the efficacy of single photon emission computed tomography/computed tomography (SPECT/CT) guided BoNT-A injections for alleviating motor and non-motor symptoms in cervical dystonia (CD) patients. It also assesses the accuracy of SPECT/CT muscle imaging in identifying responsible muscles and analyzes the quantitative significance of the SUVmax index.

**Methods:**

A total of 102 patients diagnosed with primary cervical dystonia (CD) were recruited and assessed at baseline, as well as at 2, 4, 8, 12, and 24 weeks post-botulinum toxin A (BoNT-A) injection. Among these patients, 79 underwent SPECT/CT imaging (SPECT/CT group), and 23 did not (control group). Both groups received BoNT-A injections guided by EMG, and their symptom improvements were compared. In the SPECT/CT group, the distribution of responsible muscles and its correlation with clinical phenotypes were analyzed. Furthermore, in a subset of 51 CD patients, overall and mean SUVmax of responsible muscles were quantified, and their correlation with the severity of motor symptoms was analyzed.

**Results:**

In both the Tsui and TWSTRS scales, the SPECT/CT group (*n* = 69) showed significantly higher overall improvement rates compared to the control group (*n* = 23), with statistically significant differences. In 79 CD patients, SPECT/CT imaging showed that common responsible muscles were the splenius capitis (SPCa), levator scapulae (Lev), splenius cervicis (SPCe), semispinalis capitis (SmCa), obliquus capitis inferior (OCI), and semispinalis cervicis (SSc). The deflection in CD patients was mainly influenced by the ipsilateral SPCa, SPCe, Lev, OCI, longissimus cervicis (LGc), and contralateral sternocleidomastoid muscle (SCM). In 51 CD patients, significant positive correlations were found between overall SUVmax of responsible muscles and motor symptom scores, as well as between the mean SUVmax of responsible muscles and motor symptom scores.

**Conclusion:**

SPECT/CT muscle imaging significantly improves motor symptoms in CD patients when used for BoNT-A injections. It effectively identifies responsible muscles, and its SUVmax value serves as an objective indicator of motor symptom severity. SPECT/CT shows promise as a valuable tool for diagnosing and treating CD.

## Introduction

Cervical dystonia(CD) is a focal dystonia disorder characterized by persistent or intermittent involuntary contraction of the neck muscles, leading to abnormal neck posture and/or repetitive movements [1]. Clinically, patients are categorized into four postural types: torsion, lateral inclination, forward flexion and backward extension. In practice, patients often exhibit combinations of these postures, with some also showing tremor [2].

Current treatments for CD include oral medications, botulinum toxin A (BoNT-A) injections, and surgery. Due to limited efficacy and side effects of oral drugs, and the high risks of surgery, BoNT-A injections—effective, simple to administer, and with minimal adverse reactions—have become the preferred treatment. However, BoNT-A efficacy varies widely across studies, possibly due to patient tolerance or inaccurate muscle selection. To minimize BoNT-A tolerance, injections should be spaced at least 3 months apart [3]. Traditionally, target muscle selection relied on clinical evaluation. Given CD’s diverse manifestations and complex neck muscle structure, this approach is insufficient. Pre-injection assessment of target muscles and their spasticity severity is essential for effective treatment planning. Accordingly, various guidance techniques, such as electromyography(EMG), ultrasound(US), computed tomography(CT), magnetic resonance imaging(MRI) and positron emission tomography/computed tomography(PET/CT), are used to enhance BoNT-A injection efficacy.

EMG can identify dystonic muscles but risks vascular and nerve damage and cannot detect deep muscles. US provides a clear view of neck muscle anatomy, being safe, non-invasive, time-efficient, and repeatable, yet it does not reflect muscle metabolism [4]. CT and MRI detect enlarged abnormal muscles; however, CT has limited repeatability due to radiation, and MRI is unsuitable for patients with metal implants. Studies show PET/CT-guided BoNT-A injections are more effective than clinical evaluation or EMG alone for selecting target muscles in CD [5]. However, PET/CT is expensive and not routine.

Single photon emission computed tomography/computed tomography (SPECT/CT) is a nuclear medicine imaging technique that provides both structural and functional metabolic information. ^99m^Technetium-sestamibi(^99m^Tc-MIBI) is an imaging agent used in SPECT/CT, which binds to mitochondria and reflect mitochondrial function [6]. Abnormal skeletal muscle contractions increase local oxygen and energy consumption, leading to increased blood flow and enhanced mitochondrial activity [7–9]. Based on this principle, SPECT/CT was introduced in this study to guide BoNT-A injections for CD. The application of SPECT/CT muscle imaging aims to enhance the precision and efficacy of BoNT-A injections. At present, few studies exist on SPECT/CT for CD. In 2017, Chen et al. demonstrated its feasibility in CD diagnosis and treatment [10]. Their team found SPECT/CT had 93.2% sensitivity and 88.5% specificity for detecting target muscles, comparable to PET/CT [11]. In 2020, Feng et al. conducted a double-blind randomized trial on SPECT/ CT-guided BoNT-A injection for CD. The target muscles were selected by SPECT/CT or clinical evaluation, and both groups received EMG-guided BoNT-A injections. Results showed that the SPECT/CT group had a lower repeat injection rate and longer interval compared to the clinical evaluation group [12]. In 2021, Teng et al. expanded the sample size, confirming better efficacy of SPECT/CT-guided injections over clinical evaluation alone [13].

Maximum standardized uptake value(SUVmax) is a semi-quantitative index of 18F-fluorodeoxyglucose (^18^F-FDG) PET/CT [14, 15], calculated as: SUVmax= maximum radiation concentration of the lesion (kBq/ml)/injection dose (MBq)/body weight (kg). It quantifies tissue uptake to assess disease activity. Lee et al. found SUVmax in CD correlated with Toronto Western Spasmodic Torticollis Rating Scale(TWSTRS) scores [15]. Kwon et al. showed that SUVmax of PET/CT can guide BoNT-A injections for improved outcomes in CD [16]. With the advancement of SPECT/CT technology, the application of SUVmax values in SPECT/CT has become increasingly mature, but few studies have applied SUVmax to CD patients.

Based on previous studies, this study further expanded the sample size to investigate the efficacy and benefit of SPECT/CT muscle imaging-guided BoNT-A injection in CD. Additionally, it studied the accuracy of SPECT/CT muscle imaging in identifying responsible muscles and further analyzed the quantitative significance of the SUVmax index.

## Materials and methods

### Research object

This study is a pre-planned sub-study of “The Primary Dystonia Cohort Study”. “The Primary Dystonia Cohort Study” is a multicenter, longitudinal research initiative led by West China Hospital of Sichuan University, designed to systematically investigate multiple dimensions of primary dystonia.The initial ethical approval was completed at the West China Hospital in 2017. In 2020, the Second Affiliated Hospital of Soochow University joined as a collaborating unit, and completed our local institutional review board (IRB) review and approval for the cohort study in June 2020 (Approval Number: JD-LK-2020-021-01) .This local approval served as the ethical foundation for all our subsequent research activities within the cohort framework.

The study prospectively enrolled patients with CD admitted to the Second Affiliated Hospital of Soochow University from September 2020 to September 2022. In the SPECT/CT group, target muscles were identified by integrating SPECT/CT findings with clinical assessment (including posture analysis and palpation of muscle activity), followed by EMG confirmation before injection. In contrast, BoNT-A injections for the control group were administered under EMG guidance solely based on clinical assessments.

#### Inclusion criteria

(1) The diagnostic criteria for primary CD were consistent with the “Chinese Expert Consensus on the Treatment of dystonia” of the Neurology Branch of the Chinese Medical Association in 2020 [17]; (2) Patients were aged 18–80 years; (3) Patients voluntarily agreed to undergo BoNT-A injection therapy and signed an informed consent form; (4) No intracranial organic lesions were identified in prior imaging examinations.

#### Exclusion criteria

(1) Patients with CD caused by psychogenic, drug-induced, toxic, traumatic, neoplastic, inflammatory, congenital, or other neurological factors; (2) Patients contraindicated for BoNT-A injections(e.g., amyotrophic lateral sclerosis, myasthenia gravis, Lambert-Eaton syndrome) [18]; (3) Patients using drugs that enhance BoNT-A effects(e.g., aminoglycosides, cholinesterase inhibitors, calcium channel blockers); (4) Patients who received BoNT-A within the past three months; (5) Pregnant or breastfeeding women; (6) Patients with skin infections or lesions at the injection site; (7) Patients with cognitive or psychiatric disorders; (8) Patients with prior surgery(e.g., peripheral nerve resection, deep brain stimulation, ablative procedures targeting deep brain nuclei); (9) Patients allergic to BoNT-A.

### Methods and contents of data collection

The basic information of enrolled patients was collected, including age, gender, body mass index(BMI), age at onset, disease duration, and botulinum toxin injection dose.The severity of motor symptoms was evaluated using the Tsui scale and TWSTRS Part I.

Patients were instructed to stop taking oral medication one day before SPECT/CT examination A dose of 740MBq (20mCi) of ^99m^Tc-MIBI was injected intravenously one hour before image acquisition [11, 12]. The tracer was provided by the Suzhou Branch of Shanghai Xinke Pharmaceutical Co. Ltd. After injection, patients rested quietly and avoided neck movements. For patients with tremors, a band stabilized their heads to minimize movement during imaging. The system recorded the patient’s weight, injection timing, syringe radiation activity before and after administration, and machine operation time. Imaging was performed using a SIEMENS Symbia Intevo Bold dual-probe SPECT/CT system, followed by cervical and shoulder tomography fusion.

SPECT parameters: Low energy high resolution collimator, energy peak 140KeV, window width 15%, matrix 256 × 256, magnification 1.0, 360° tomography acquisition, 6°/frame, 20s/frame. CT scan: Layer thickness 2 mm, matrix 512 × 512, tube voltage 130Kv, reference tube current 120mAs.Image fusion: SIEMENS NM-CT fusion software was used to perform SPECT and CT tomography image fusion processing.

### Judgment of responsible muscle

**Fig. 1:**
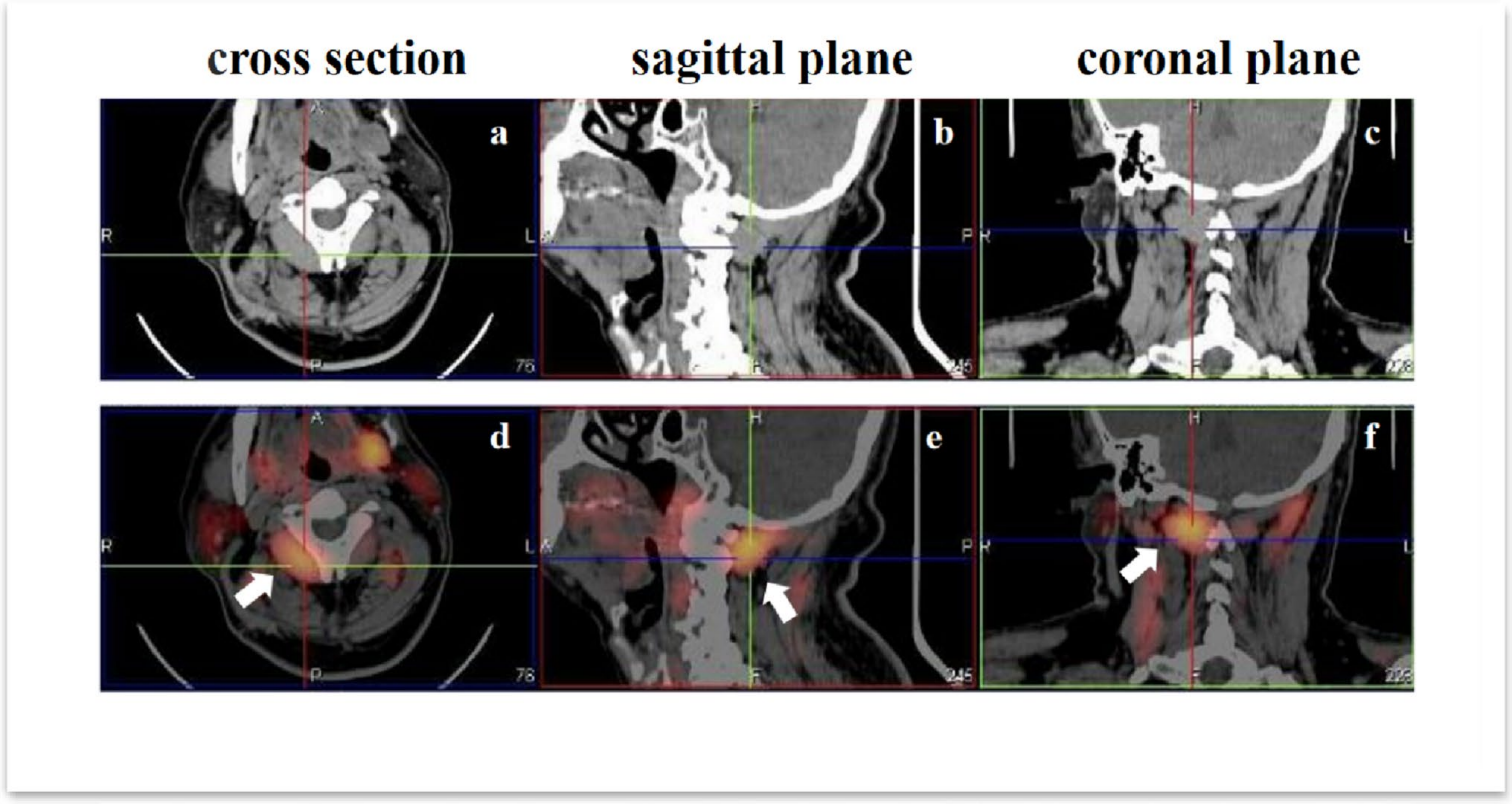
A case of ^99m^Tc-MIBI SPECT/CT muscle imaging. Note: Figures a, b, and c are CT scan images for muscle localization, and Figure d, e, and f are SPECT/CT fusion imaging images. The highly concentrated ^99m^Tc-MIBI region pointed by the arrow was observed at this level, integrating orientation of cross section, sagittal plane and coronal plane, and the region was in line with the right obliquus capitis inferior located in CT tomography images, and then the right obliquus capitis inferior was identified as one of the responsible muscles of the patient.

The image slices were interpreted by the same senior nuclear medicine physician, who was blinded to the clinical symptoms of all patients with CD. In the SPECT/CT tomography fusion image, the region of high ^99m^Tc-MIBI uptake corresponded to the anatomical location of a specific neck muscle identified on the CT tomography image, confirming this muscle as the responsible muscle(Fig. 1).

### Determination of overall SUVmax value

The Region of interest(ROI) of the neck muscle group was delineated on the cross-sectional fusion image with a threshold set at 50% of the maximum value. The Symbia Intevo bold SPECT/CT software automatically generates the Volume of interest(VOI). Subsequently, the VOI boundaries were meticulously adjusted on cross-sectional, sagittal, and coronal images to ensure comprehensive inclusion of all neck muscles. Finally, the overall SUVmax value was calculated by X-SPECT quantitative software.

### Determination of mean SUVmax value

On the cross-sectional fusion image, the ROI for the responsible neck muscle was outlined layer by layer with a threshold set at 50% of the maximum value, and the VOI was automatically generated. The SUVmax value for each responsible neck muscle was calculated by X-SPECT quantitative software. The average SUVmax value for the responsible muscles was determined as follows: (SUVmax1 + SUVmax2 + SUVmax3 + … + SUVmaxN)/N, where N represents the total number of responsible muscles identified per patient.

### EMG guided BoNT-A injection therapy

BoNT-A(Hengli, 100U/vial, National Drug Approval No.:S10970037) was provided by Lanzhou Institute of Biological Products. It was diluted with 0.9% saline to a concentration of 6 ml per 100 U. Injection doses ranged from 150 to 400U, depending on the patient’s condition and weight. Electromyography was performed using a KEYPOINT instrument(Dantec, Denmark). All BoNT-A injections were administered by the same neurologist certified in botulinum toxin injections. During the procedure, patients were seated comfortably in an upright position with their arms hanging naturally at their sides. An EMG needle was inserted into the target muscle, which was determined either by examination results in the SPECT/CT group or clinical assessment in the control group. EMG guidance was selected to confirm abnormal spontaneous activity in target muscles, complementing the anatomical and metabolic information from SPECT/CT. Muscles showing involuntary motor unit potentials(MUPs) at rest were identified as dystonic, and appropriate dose of BoNT-A was determined based on the amplitude of the MUPs.

### Follow-up Assessment and Efficacy Evaluation

The same trained neurologist evaluated the scales and conducted follow-up assessments at baseline, 2, 4, 8, 12, and 24 weeks post-treatment. If a patient received a second botulinum toxin injection within 6 months, follow-up was discontinued, and the interval between injections was recorded. Improvement rate=(total score before treatment-total score after treatment)/total score before treatment ×100% [19]. Time interval of repeated injection: The time interval between two consecutive BoNT-A injections was expressed in “day”. No secondary injections were administered during the 24-week follow-up period, and the time interval for repeated injection was therefore recorded as 180 days.

### Statistical method

Quantitative data that followed a normal distribution were expressed as mean ± SD, independent-sample t-tests was used between groups, and Pearson correlation analysis was conducted to evaluate correlations. Quantitative data with non-normal distribution were represented by median(quartile method), non-parametric rank sum test(Mann-Whitney U test) was used between groups, and Spearman correlation analysis was performed for correlation. Qualitative data were presented as composition ratios, and intergroup comparisons were conducted using the Chi-square test. The differences in motor symptoms between the SPECT/CT group and the control group at each time point were assessed by two-factor repeated measure ANOVA, and the data were expressed as mean ± SE. A p-value less than 0.05 was considered statistically significant.

## Results

### Therapeutic efficacy of SPECT/ CT-guided BoNT-A injection in CD

This study included 102 patients with CD, 79 in the SPECT/CT group and 23 in the control group. No significant differences were found between the groups in gender, age, onset age, disease duration, BMI, botulinum toxin dose, or injection frequency(*p* > 0.05). The mean re-injection interval was 174.8 ± 14.9 days in the SPECT/CT group and 160.5 ± 28.0 days in the control group. The SPECT/CT group had a significantly longer interval(*p* = 0.027)(Table 1).

**Table 1:** General clinical information

Baseline data	SPECT/CT group (*n* = 79)	Control group (*n* = 23)	*p*
Gender(Man, %)	38.00	26.09	0.294
Age(Year)	46.96 ± 13.41	49.39 ± 12.41	0.439
Age of onset(Year)	43.04 ± 14.13	46.09 ± 10.59	0.340
Course of disease (Mouth)	47.71 ± 81.21	38.96 ± 46.88	0.623
BMI(kg/m²)	23.18(20.76, 24.97)	23.31(20.76, 23.80)	0.854
Injection dose(U)	200(200, 250)	200(200, 300)	0.829
Injection times	1(1, 2)	1(1, 2)	0.174
Re-injection interval(Day)	174.77 ± 14.88	160.52 ± 28.00	0.027^*^

Note: Quantitative data are expressed as mean ± SD for normal distributions and as median (IQR) for non-normal distributions. Qualitative data are described using proportions (%). BMI: Body mass index=weight(kg)/height(m²). *p < 0.05

There were 69 patients in the SPECT group and 23 in the control group who completed 24 weeks follow-up. Baseline scores for both scales showed no significant differences between groups(*p* > 0.05). For the Tsui scale, significant differences were found at 8 weeks(*p* = 0.005) and 12 weeks(*p* = 0.019), but not at 2, 4, or 24 weeks(*p* > 0.05). For the TWSTRS scale, no significant differences were observed at any time point(*p* > 0.05).

**Fig. 2:**
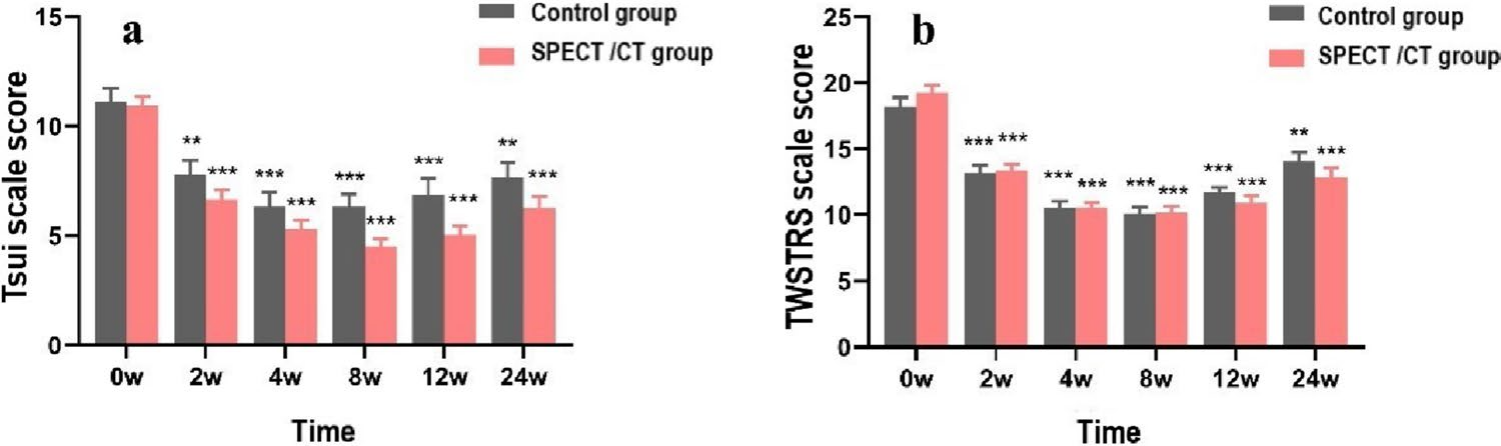
Comparison of motor symptom scale between SPECT/CT group and control group. Note: Differences between SPECT/CT and control groups at each time point were assessed by two-factor repeated-measures ANOVA. Data are mean ± SE. *Indicates statistical difference from baseline(*p < 0.05, **p < 0.01, ***p < 0.001). SPECT/CT group: *n* = 69; Control group: *n* = 23

Changes in the Tsui and TWSTRS scores for the SPECT/CT and control groups were analyzed using two-factor repeated-measures ANOVA. For the Tsui scale, the follow-up scores of both groups decreased significantly compared to baseline across all time points(Fig. 2a: F_group_(1,535) = 12.570, *p* < 0.001; F_time_(5,535) = 24.100, *p* < 0.001). For the TWSTRS scale, the follow-up scores of both groups also decreased significantly compared to baseline at all time points (Fig. 2b: F_group_(1,536) = 0.070, *p* = 0.792; F_time_(5,536) = 42.810, *p* < 0.001).

**Fig. 3:**
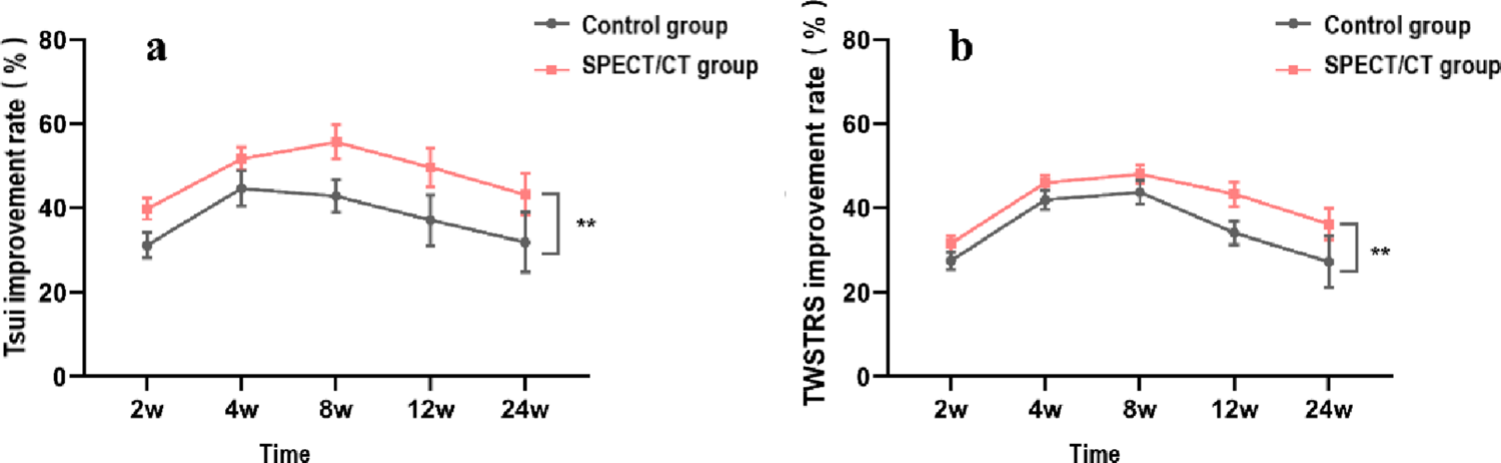
Comparison of motor symptom improvement rate between SPECT/CT group and control group. Note: Differences between SPECT/CT and control groups at each time point were analyzed using two-factor repeated-measures ANOVA. Data are mean ± SE. *Significant differences between groups, **p < 0.01. SPECT/CT group: *n* = 69; Control group: *n* = 23

Two-factor repeated-measures ANOVA was used to analyze the improvement rates of the SPECT/CT and control groups at each follow-up time point. For the Tsui scale, the SPECT/CT group had a higher improvement rate than the control group (Fig. 3a: F_group_(1,450) = 9.990, *p* = 0.002; F_time_(4,450) = 2.768, *p* = 0.027). Similarly, for the TWSTRS scale, the SPECT/CT group showed a higher improvement rate (Fig. 3b: F_group_(1,450) = 7.737, *p* = 0.006; F_time_(4,450) = 8.679, *p* < 0.001).

### Diagnostic value of SPECT/CT muscle imaging in CD

Clinically, the common responsible muscles in CD patients include the sternocleidomastoid(SCM), splenius capitis(SPCa), splenius cervicis(SPCe), trapezius(TPZ), levator scapulae(Lev), semispinalis cervicis(SSc), semispinalis capitis(SmCa), spinalis cervicis (SPc), obliquus capitis inferior(OCI), scalenus medius(ScM), longus capitis(LC), longus colli(Lc), and longissimus cervicis(LGc). For each patient, 13 types of muscles mentioned above were selected for the determination of the responsible muscle, and a total of 26 muscles on both sides were included. Therefore, a total of 2054 muscles from 79 patients were included for responsible muscle determination.

SPECT/CT muscle imaging in 79 patients revealed the following distribution of responsible muscles: SCM involvement in 40 cases, SPCa in 62 cases, SPCe in 54 cases, TPZ in 41 cases, Lev in 58 cases, SSc in 50 cases, SmCa in 53 cases, SPc in 38 cases, OCI in 50 cases, ScM in 36 cases, LC in 44 cases, Lc in 29 cases, and LGc in 42 cases. Notably, the muscles with higher involvement frequencies included SPCa(78.48%), Lev(73.42%), SPCe(68.35%), SmCa(67.09%), OCI(63.29%), and cervical SSc(63.29%) (Fig. 3).

**Fig. 4:**
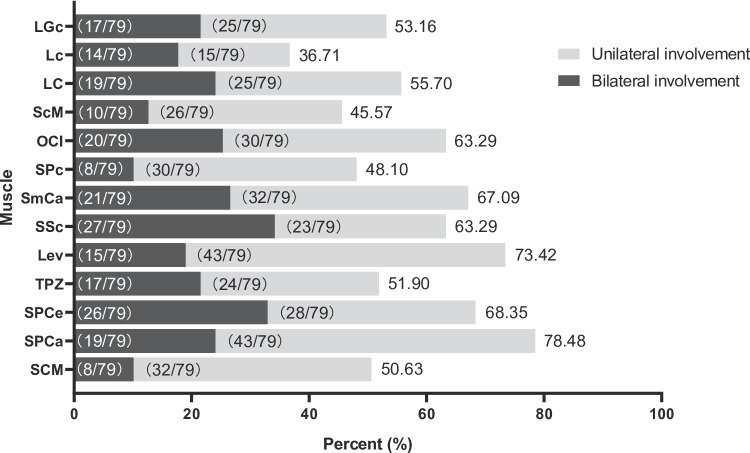
Distribution of responsible muscles in SPECT/CT muscle imaging in 79 CD patients. Note: SCM: Sternocleidomastoid muscle, SPCa༚Splenius capitis, SPCe༚Splenius cervicis, TPZ༚Trapezius, Lev༚Levator scapulae, SSc༚Semispinalis cervicis, SmCa༚Semispinalis capitis, SPc༚Spinalis cervicis, OCI༚Obliquus capitis inferior, ScM༚Scalenus medius, LC༚Longus capitis, Lc༚Longus colli, LGc༚Longissimus cervicis.

SSc and SPCe demonstrated bilateral involvement at a higher frequency, while Lev, SPCa, SCM, and SmCa exhibited predominantly unilateral involvement. In this study, for the deep muscles that were not frequently examined during previous injections, such as OCI, LC, LC, and LGc, SPECT/CT muscle imaging showed that their examination frequencies were significantly high, at 63.29%, 55.70%, 36.71%, and 53.16% respectively (Fig. 4).

Among the 79 patients, there were 4 cases of simple torsion, 4 cases of simple lateral inclination, 41 cases of torsion + lateral inclination, 26 cases of torsion + lateral inclination + forward flexion, and 4 cases of torsion + lateral inclination + backward extension. Due to the complexity of clinical manifestations, the 79 patients were divided into a left-leaning group(*n* = 45) and a right-leaning group(*n* = 34) according to head and neck deviation. The results showed no significant correlation between head and neck deviation and unilateral or bilateral responsibility muscle involvement in CD patients(*p* > 0.05). In cases of unilateral involvement only, there was a significant correlation between the side of involvement of SCM, SPCa, SPCe, Lev, OCI, and LGc and head and neck deviation(*p* < 0.05). Head and neck deviation in CD patients is primarily caused by the combined action of ipsilateral SPCa, SPCe, Lev, OCI, LGc, and contralateral SCM(Table 2).

**Table 2:** Correlation analysis of responsible muscle distribution and head and neck deviation in SPECT/CT muscle imaging

Muscle	Head and neck deviation	Unilateral involvement					Bilateral involvement	*p*_2_
		Left side	Right side		Total	*p*_1_		
SCM	L	9		9	18	0.019^*^	3	0.580
	R	13		1	14		5	
SPCa	L	18		4	22	0.000^*^	13	0.206
	R	4		17	21		6	
SPCe	L	10		3	13	0.007^*^	17	0.161
	R	3		12	15		9	
TPZ	L	8		5	13	0.885	13	0.258
	R	8		3	11		4	
Lev	L	14		9	23	0.040^*^	10	0.559
	R	5		15	20		5	
SSc	L	6		7	13	1.000	16	0.845
	R	5		5	10		11	
SmCa	L	9		12	21	0.798	14	0.938
	R	6		5	11		7	
SPc	L	10		9	19	0.919	6	0.843
	R	6		5	11		2	
OCI	L	9		4	13	0.025^*^	11	0.419
	R	4		13	17		9	
ScM	L	13		4	17	0.230	6	1.000
	R	4		5	9		4	
LC	L	7		8	15	0.137	10	0.625
	R	1		9	10		9	
Lc	L	6		2	8	0.201	9	0.825
	R	2		5	7		5	
LGc	L	10		5	15	0.041^*^	9	0.650
	R	2		8	10		8	

Note: For total cases ≥ 40 and all theoretical frequencies ≥ 5, p is the Pearson chi-square result. If ≥ 40 cases include one theoretical frequency ≥ 1 and < 5, p is the continuity correction result. If ≥ 40 cases have at least two theoretical frequencies ≥ 1 and < 5, or if total cases are < 40 or any frequency < 1, p is the Fisher’s exact test result.*p < 0.05. p1 represents the statistical significance of the difference between the left and right sides in cases of unilateral involvement, while p2 indicates the statistical significance of the difference between unilateral and bilateral involvement.L: left-leaning group; R: right-leaning group. SCM: Sternocleidomastoid muscle, SPCa༚Splenius capitis, SPCe༚Splenius cervicis, TPZ༚Trapezius, Lev༚Levator scapulae, SSc༚Semispinalis cervicis, SmCa༚Semispinalis capitis, SPc༚Spinalis cervicis, OCI༚Obliquus capitis inferior, ScM༚Scalenus medius, LC༚Longus capitis, Lc༚Longus colli, LGc༚Longissimus cervicis

Among the 79 CD patients, they were divided into the tremor group(*n* = 45) and the non-tremor group(*n* = 34). The statistical results showed no significant difference in unilateral or bilateral muscle involvement between the two groups (*p* > 0.05)(Table 3).

**Table 3:** Correlation analysis between the distribution of responsible muscles and the tremor phenotype in SPECT/CT muscle imaging

Muscle		Unilateral involvement	Bilateral involvement	Total	*p*
SCM	T	20	6	26	0.804
	N-T	12	2	14	
SPCa	T	26	8	34	0.180
	N-T	17	11	28	
SPCe	T	18	16	34	0.835
	N-T	10	10	20	
TPZ	T	13	10	23	0.767
	N-T	11	7	18	
Lev	T	22	8	30	0.885
	N-T	21	7	28	
SSc	T	10	17	27	0.168
	N-T	13	10	23	
SmCa	T	16	13	29	0.394
	N-T	16	8	24	
SPc	T	19	3	22	0.189
	N-T	11	5	16	
OCI	T	18	11	29	0.726
	N-T	12	9	21	
ScM	T	17	6	23	0.763
	N-T	9	4	13	
LC	T	13	10	23	0.967
	N-T	12	9	21	
Lc	T	9	9	18	0.812
	N-T	6	5	11	
LGc	T	12	13	25	0.065
	N-T	13	4	17	

Note: For total cases ≥ 40 and all theoretical frequencies ≥ 5, *p* is the Pearson chi-square result. If ≥ 40 cases include one theoretical frequency ≥ 1 and < 5, *p* is the continuity correction result. If ≥ 40 cases have at least two theoretical frequencies ≥ 1 and < 5, or if total cases are < 40 or any frequency < 1, *p* is the Fisher’s exact test result.*p < 0.05. T: Tremor group; N-T: Non-tremor group

### Quantitative significance of SUVmax index in SPECT/CT muscle imaging

In this study, SUVmax values were successfully obtained from 51 out of 79 CD patients who underwent SPECT/CT examination. Among these 51 CD patients, overall SUVmax value of the total neck muscle group was 2.64 ± 0.75, and mean SUVmax value of the responsible muscle was 2.05 ± 0.54. The other general clinical data, such as gender, age, age of onset and course of disease, were summarized in Table 4.

**Table 4:** General clinical data in 51 CD patients

Baseline data	Statistical result(*n* = 51)
Gender(Man, %)	35.3
Age(Year)	45.20 ± 11.87
Onset of age(Year)	41.00 ± 13.66
Course of disease(Mouth)	50.64 ± 91.85
Overall SUVmax	2.64 ± 0.75
Mean SUVmax	2.05 ± 0.54

Note: Quantitative data are expressed as mean ± SD for normal distributions. Qualitative data are described using proportions (%). BMI=weight(kg)/height(m²). *p < 0.05

**Fig. 5:**
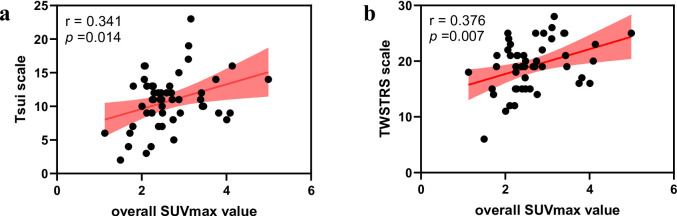
Correlation analysis between overall SUVmax value and motor symptom score in 51 CD patients. Note: Pearson correlation analysis was performed. Statistical significance was set at *p* < 0.05

Pearson correlation analysis was conducted between overall SUVmax value of the total neck muscle and the motor symptoms scores. The results showed that overall SUVmax value was significantly correlated with both the Tsui(*p* = 0.014, *r* = 0.341) and TWSTRS scale scores(*p* = 0.007, *r* = 0.376), indicating a positive correlation(Fig. 5).

**Fig. 6:**
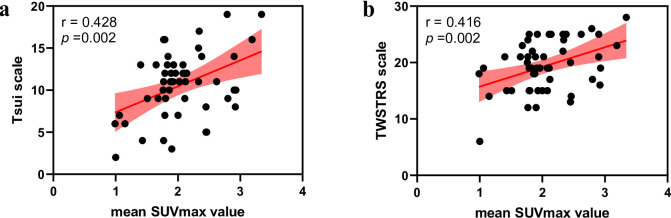
Correlation analysis between mean SUVmax of responsible muscle and motor symptom score in 51 CD patients. Note: Pearson correlation analysis was performed. Statistical significance was set at *p* < 0.05

The SUVmax values of 578 responsible muscles from 51 patients were measured. Pearson correlation analysis was conducted between the mean SUVmax value of the responsible muscle and the motor symptom scores in each CD patient. The results showed that mean SUVmax value of the responsible muscle was significantly positively correlated with both the Tsui scale(*r* = 0.428, *p* = 0.002) and the TWSTRS scale scores(*r* = 0.416, *p* = 0.002)(Fig. 6).

## Discussion

This study showed that CD patients improved significantly in motor symptoms with or without SPECT/CT-guided BoNT-A injections. Effects lasted up to 24 weeks. SPECT/CT guidance improved efficacy and extended the re-injection interval, consistent with prior studies [11–13]. Uneven ^99m^Tc-MIBI uptake in CD muscles allowed precise localization of pathogenic points, enhancing injection accuracy. However, follow-up was limited to six months, with a maximum re-injection interval of 180 days. Longer follow-up is needed to confirm actual re-injection intervals.

Among 79 patients with CD, 6 muscle groups showed notably high involvement rates based on SPECT/CT localization: SPCa, Lev, SPCe, SmCa, OCI, and SSc. It’s suggested that clinicians should prioritize these muscles for BoNT-A injections in CD patients without SPECT/CT examination and consider routine EMG evaluation. Traditionally, the SCM is the primary target for BoNT-A treatment [20]. This study revealed that the involvement frequency of the SCM was 50.63%. Compared to muscles with higher involvement rates, SCM is not the main contributor to CD, which challenges traditional perspectives. In 2011, Reichel et al. proposed the “COL-CAP” concept to enhance the clinical classification of CD [21]. Anatomically, C2 serves as a demarcation point. Dystonia in the muscles between the skull and C2, which control head postural movement, results in abnormal head posture relative to the neck, termed “Caput”. Dystonia in the muscles controlling neck postural movement between C2-C7 leads to abnormal neck posture relative to the torso, termed “Collum”. SCM originates from the manubrium sterni and the sternal end of the clavicle, inserting into the mastoid process of the temporal bone, In dystonia, abnormal SCM activity predominantly manifests as “Caput” type involvement. Current studies indicate that “Collum” is the most prevalent subtype of CD [22]. Given the “COL-CAP”, the relatively low frequency of SCM involvement observed in this study can be reasonably explained.

OCI is the largest suboccipital muscle. Under physiological conditions, its role in head rotation is minimal, but in CD, it generates strong ipsilateral head rotation. LC and Lc are deep prevertebral muscles responsible for head flexion. LGc, as part of the erector spinae muscle group, supports the head during forward inclination and assists in neck flexion [23]. These four muscles belong to the deep neck musculature. Due to their proximity to blood vessels and nerves, EMG-guided detection and injection are technically challenging and risky. In prior CD treatments, these muscles were rarely targeted unless identified as causative factors. SPECT/CT imaging in this study revealed non-negligible involvement rates of the OCI, LC, Lc, and LGc at 63.29%, 55.70%, 36.71%, and 53.16%, respectively. Similar to prior FDG PET studies, SPECT/CT identified hypermetabolic deep muscles that were not routinely evaluated or injected due to technical and safety limitations. This may have contributed to suboptimal treatment responses in some patients and highlights the potential opportunity in translating imaging findings into injectable targets.

Muscles involved in CD can be categorized into three groups: dystonic muscles, “antagonistic muscles,” and “compensatory muscles”. Dystonic muscles are primarily responsible for abnormal head and neck postures and serve as the main targets for BoNT-A injection therapy. “Antagonistic muscles” are passively stretched or contracted due to abnormal postural movements, producing opposing postural movements or tremors; in some cases, these muscles may also require limited BoNT-A injections. “Compensatory muscles” are voluntarily activated by CD patients to correct abnormal postures and typically do not require BoNT-A treatment [11]. In this study, it was found that the deflection direction in CD patients was primarily influenced by the ipsilateral SPCa, SPCe, Lev, OCI, LGc, and the contralateral SCM. The ipsilateral muscles responsible for head and neck deviation in CD patients are predominantly spontaneous dystonia muscles, which can typically be managed with BoNT-A injections. The contralateral SCM may serve as the “antagonist muscle” due to passive tension, and BoNT-A treatment can be considered based on the individual condition of the patient.

This study found no significant difference in muscle distribution between tremor and non-tremor CD patients. Current research shows that basal ganglia and cerebellar dysfunction, along with their interconnected cortical and subcortical structures, contribute to CD pathogenesis, with cerebellar issues being a key factor in tremor [24]. Laura Avanzino et al. investigated the tactile and proprioceptive differences between CD patients with and without tremor. Their findings revealed that both CD subgroups exhibited a significantly higher tactile resolution threshold than the healthy control group. Additionally, proprioceptive acuity was enhanced in tremor CD compared to healthy controls and non-tremor CD [25]. Another study showed that tremor CD had more severe systemic ataxia than non-tremor CD [26]. A study involving 188 patients with neck and segmental dystonia collected clinical and cranial imaging data, revealing cerebellar atrophy in 17(9%) of the participants. Among these 17 patients, 14(82.4%) were diagnosed with CD, including 11(78.6%) with the tremor subtype. However, the study did not establish whether cerebellar atrophy is more severe in tremor CD compared to non-tremor CD [27]. Based on the aforementioned studies, it is reasonable to hypothesize that CD tremor originates from central lesions, referred to as the “essence”, while muscle involvement represents only the peripheral manifestation, termed the “appearance”. This explains the lack of significant differences in peripheral muscle involvement between the tremor and non-tremor groups. Nevertheless, further research is required to elucidate the specific central lesion location associated with tremor CD.

Previous studies have indicated that the SUVmax of the responsible muscle in ^18^F-FDG PET/CT could serve as a guiding parameter for BoNT-A injection therapy, leading to favorable therapeutic outcomes in CD patients [16]. To date, no studies have investigated SUVmax values from SPECT/CT in CD. This study found that both the overall SUVmax of neck muscles and the mean SUVmax of responsible muscles significantly correlate with CD motor symptom severity, consistent with PET/CT results. These findings suggest SPECT/CT SUVmax values can objectively reflect CD motor symptom severity. The abnormal contraction of responsible muscles in CD increases oxygen and energy consumption in local muscles, leading to a compensatory increase in blood flow and mitochondrial function [7]. This, in turn, enhances ^99m^Tc-MIBI uptake by responsible muscles, as reflected by the increased objective index of SUVmax [9]. We reasonably hypothesized that the SUVmax value of the responsible muscle could serve as a guiding index for determining the injection dose of BoNT-A. Specifically, a higher SUVmax value of the responsible muscle may indicate that an appropriately increased injection dose of BoNT-A could lead to better therapeutic outcomes. Generally, the correlation coefficient between the mean SUVmax value of the responsible muscle and the severity of motor symptoms was greater than that of the overall SUVmax value of total neck muscle. This suggests that the mean SUVmax value of the responsible muscle more accurately reflects the severity of motor symptoms. The overall SUVmax value of the total neck muscle is heavily influenced by its maximum value, whereas obtaining the mean SUVmax of the responsible muscle involves a more complex process. Both indices possess distinct advantages and limitations, and they can complement each other in practical applications. In summary, SPECT/CT muscle imaging for BoNT-A injection in CD demonstrates significant potential and promising prospects.

While SPECT/CT provides valuable metabolic and structural information, its cost and limited availability may restrict routine use. However, in patients with complex or refractory CD, SPECT/CT could be a cost-effective tool by improving injection accuracy, prolonging treatment intervals, and reducing cumulative BoNT-A doses. Future health-economic studies are needed to evaluate its cost–benefit ratio in different healthcare settings.

However, our article still has certain limitations. This study did not systematically compare the diagnostic accuracy of SPECT/CT versus clinical assessment on a per-muscle basis using EMG as a gold standard. Future studies incorporating such a design would further clarify the added value of SPECT/CT in muscle identification.

## Conclusion

The application of SPECT/CT muscle imaging in BoNT-A injection for CD enhances motor symptom improvement. It identifies responsible muscles in CD patients, with SUVmax providing an objective measure of symptom severity. SPECT/CT muscle imaging is a promising tool for diagnosing and treating CD.

## Data Availability

The data that support the findings of this study are available from the corresponding author upon reasonable request.
